# GGPPS1 predicts the biological character of hepatocellular carcinoma in patients with cirrhosis

**DOI:** 10.1186/1471-2407-14-248

**Published:** 2014-04-09

**Authors:** De-cai Yu, Jia Liu, Jun Chen, Jiao-jiao Shao, Xiao Shen, Hong-guang Xia, Chao-jun Li, Bin Xue, Yi-tao Ding

**Affiliations:** 1 Department of Hepatobiliary Surgery, the Affiliated Drum Tower Hospital, Medical School of Nanjing University, Nanjing, Jiangsu Province 210008, P.R. China; 2 Institute of Hepatobiliary Surgery, Nanjing University, Nanjing, Jiangsu Province 210008, P.R. China; 3 Jiangsu Key Laboratory of Molecular Medicine of the School of Medicine, Nanjing University, National Resource Center for Mutant Mice and MOE Key Laboratory of Model Animals for Disease Study, Model Animal Research Center, Nanjing, Jiangsu Province 210093, P.R. China; 4 Department of Cell Biology, Harvard Medical School, Boston, MA 02115, USA

## Abstract

**Background:**

Hepatocellular carcinoma (HCC) has been associated with diabetes and obesity, but a possible connection with the metabolic syndrome (MetS) and its potential interaction with hepatitis and cirrhosis are open to discussion. Our previous investigations have shown that GGPPS1 plays a critical role during hyperinsulinism. In this report, the expression and distribution of GGPPS1 in liver cancer, and its clinical significance were investigated.

**Methods:**

70 patients with hepatocellular carcinoma (HCC) were included in this study. Three different types of tissues from each HCC patient were assembled immediately after surgical resection: tumor-free tissue >5 cm far from tumor edge (TF), adjacent nonmalignant tissue within 2 cm (AT), and tissue from the tumor (TT). Normal liver tissues from 10 liver transplant donors served as healthy control (HC) while 10 patients with liver cirrhosis as cirrhosis control (CC). The expression and distribution of GGPPS1 were detected by immunohistochemistry, western blots, or real-time PCR. The relationship between the expression of GGPPS1 and clinic pathologic index were analyzed.

**Results:**

We found that GGPPS1 was intensified mainly in the cytoplasm of liver tumor cells. Both the expression of GGPPS1 mRNA and protein were upregulated in TT comparing to AT or TF. Meanwhile, HCC patients with cirrhosis had relative higher expression of GGPPS1. In addition, many pathologic characters show close correlation with GGPPS1, such as tumor stage, vessel invasion, and early recurrence.

**Conclusion:**

GGPPS1 may play a critical role during the development of HCC from cirrhosis and is of clinical significance for predicting biological character of HCC.

## Background

Liver cancer in men is the fifth most frequently diagnosed cancer and ranks the second cause of cancer-related death worldwide [1]. Liver cancer comprises diverse, histological distinct primary hepatic neoplasms while hepatocellular carcinoma (HCC) represents the major histological type and likely accounts for 75% cases [2]. The etiology of HCC is likely to involve interactions between multiple risk factors, including hepatitis B or C virus (HBV or HCV), hepatosteatosis, and long-term exposure to toxic chemicals, leading to liver injury and inflammation [3-5]. Persistent inflammation fosters a chronic liver disease condition that eventually culminates in liver cirrhosis. In fact, cirrhosis is present in about 80–90% of HCC patients and is thereby the largest risk factor [6]. With the recent worldwide epidemic of obesity and metabolic syndrome [7,8], it has been reported that underlying insulin resistance and fatty liver disease may potentially increase the risk and worsen HCC outcomes [9].

The lethality of liver cancer partially results from its resistance to existing anticancer agents and lack of biomarkers that can detect surgically resectable incipient nodule. Recently, improved knowledge of signaling pathways that regulate tumor cell proliferation, differentiation, angiogenesis, and metastasis has led to several possible therapeutic targets. Hence, interference of MAPK, PI3K/AKT/mTOR, WNT/b-catenin and IGF pathways are suggested to be promising therapies [10]. Additionally, Statin can decrease synthesis of downstream products in mevalonate pathway, thus benefits in the chemoprevention and management of HCC. As intermediate isoprenoid, GGPP can overcome the inhibition of COX-2 protein expression by simvastatin [11]. Additionally, it has been reported that inhibition of GGPPS induced autophagy in prostate cancer [12] and decreased breast cancer cell migration [13]. However, no study on the relationship between expression level of GGPPS and HCC biological character has been reported till date. Therefore, we hypothesized that GGPPS1 could be correlated with carcinogenesis of HCC.

Mevalonate pathway can produce isoprenoids that are vital for diverse cellular functions, ranging from cholesterol synthesis to growth control [14]. GGPPS1 is a branch point enzyme in this pathway and catalyzes the synthesis of all-trans-geranylgeranyl diphosphate (GGPP), the 20 carbon isoprene moieties post-translationally incorporated into several proteins, including many members of the Ras and Rho family [15]. According to our previous study, in the pathogenesis of cigarette smoke-related pulmonary disease and type 2 diabetes mellitus (T2DM), GGPPS1 can enhance Ras prenylation and membrane association, further leading to pulmonary inflammation and insulin resistance through MAPK pathway [16,17]. However, the contribution of GGPPS1 to HCC remains largely unexplored. Considering the critical role of these small GTPases in tumor genesis and metastasis [18-20], we propose that GGPPS1 may have involvement in the development of HCC and can be used as biomarker in diagnose.

In this study, we investigated the expression and distribution of GGPPS1 in liver tissues and tumor tissues from HCC patients. Additionally, the correlation of protein level with clinicpathologic factors was analyzed to determine whether it is an independent prognostic marker for predicting clinical outcomes of HCC in patients.

## Methods

### Patients and samples

Between January 2005 and August 2012, 70 patients with hepatocellular carcinoma (HCC) were included in this study in the Department of Hepatobiliary Surgery of Drum Tower Hospital. None of the patients had undergone any preoperative therapies. Three different types of tissues from each HCC patient were assembled immediately after surgical resection: tumor-free tissue >5 cm far from the tumor edge (TF), adjacent nonmalignant tissue within 2 cm (AT), and tissue from the tumor (TT). Areas of tissue necrosis and hemorrhage were excluded. Normal liver tissues from 10 liver transplant donors served as healthy control (HC) while 10 patients with liver cirrhosis as cirrhosis control (CC).

All of the tissue samples were snap frozen immediately after resection and kept in liquid nitrogen until they were used for experiments. A senior pathologist did histopathologic examination of all specimens with experience in HCC pathology (Dr. WU Hongyan), who was unaware of the preoperative clinical data and immunostaining results. Tumor differentiation was assessed according to Edmonson and Steiner grading system. Serial sections of the tumors and surrounding liver were examined to identify any tumor encapsulation, microscopic venous invasion, and microsatellite lesions. The degree of HCC invasiveness was verified according to the invasiveness scoring system for HCC. Tumor stage was defined according to tumor-node-metastasis (TNM) classification of the American Joint Committee on International Union against Cancer.

Preoperative clinical and laboratory, and pathological data were prospectively assembled for each patient in our HCC database. All patients were followed and monitored regularly for tumor recurrence by alpha-fetoprotein level (monthly) and chest X-ray, together with B ultrasonic or computed tomography scan (every 3 months). A diagnosis of recurrence was based on typical imaging appearance in computed tomography scan and an elevated alpha-fetoprotein level. All of the patients were followed until death or until the study closing date of January 1st, 2013.

This study was approved by the Scientific Research Ethics Committee of the affilisted Drum Tower Hospital, Medical School of Nanjing University, and an informed consent was obtained from all participants.

### Immunohistochemistry

Formalin-fixed and paraffin-embedded HCC sections with a thickness of 4 μm were dewaxed in xylene and graded alcohols, hydrated and washed in phosphate buffered saline (PBS). After pretreatment in a microwave oven, endogenous peroxidase was inhibited by 3% hydrogen peroxide in methanol for 20 min, followed by avidin-biotin blocking using a biotin-blocking kit (DAKO, Germany). Slides were then immunoassayed with the antibodies to human GGPPS1 (1:200; Proteintech), CD34 (1:300; Santa Cruz Biotechnology), VEGF_165_ (1:100, Santa Cruz), p53, PCNA, EGFR, or COX-2 (1:200; DAKO). A subsequent reaction was performed with biotin-free HRP enzyme-labeled polymer from an En Vision plus detection system (DAKO, CA). Positive reactions were visualized with diaminobenzidine (DAB) solution followed by counterstaining with hematoxylin. Negative controls were performed using non-immune goat serum instead of the primary antibodies.

Semi-quantitative IHC detection was used to determine the GGPPS1, PCNA, EGFR, or COX-2 protein levels. A brown particle in nuclei was considered as positive labeling. Immunostain was scored using a 4-point scale (0–4) system according to the intensity of staining and the percentage of positive cells. IHC evaluation was performed according to the method described before [21]. For each case, 1000 cells were randomly selected and scored. HCC sections were observed under light microscopy and the staining intensity scores were independently assessed by 2 pathologists (Dr. CHEN Jun and WU Hongyan).

### Real-time PCR

Acid guanidine thiocyanatephenol-chloroform extraction was used to isolate total RNA from liver tissues [22]. With random hexamer primers, the maximum allowed volumes of RNA samples were transcribed with ExScript RT reagent kit (TaKaRa) according to the manufacturer’s protocol. RNA samples without reverse transcription were used as negative controls.

PCR Primers and probes for human GGPPS1 and 18S genes were designed with Primer Express 2.0 software (Applied Biosystems) and synthesized by Genecore. The basic information on primers, including gene name, forward primer, reverse primer are the following: GGPPS1, CCAGGTAAACAAGTGAGAACCAA, CGTCGGAGTTTTGAGTTGTCT, and 18S, GTCTGTGATGCCCTTAGATG, AGCTTATGACCCGCACTTAC. For the amplification of GGPPS1 and 18S genes, real-time PCR was done in triplicate for each sample in a 20 μl reaction mixture, which consisted of template DNA (2 μl), primers (900 nmol/L), probe (250 nmol/L), Mg^2+^ (5 mmol/L) and Ex Taq HS (0.1 units/AL; ExScript Real-time PCR Kit, TaKaRa). PCR was done in a Stratagene Mx3005P instrument using the following thermal cycles: one cycle of 1 min at 95°C, 40 cycles of 15 s at 95°C, 15 s at 58°C and 30s at 72°C. Amplification efficiency of each individual sample was calculated by version 7.0 of LinRegPCR program (a gift from C.R.Ramakers, 3 Academic Medical Centre, University of Amsterdam, Amsterdam, the Netherlands). According to the method tested by Pfaffl, the relative expression ratio of a targeted gene was calculated based on efficiency and the Ct compared with a reference gene (18S).

### Western blots

Tissue lysates were boiled with 6 × sodium dodecyl sulfate (SDS) loading buffer and then fractionated by SDS-PAGE. The proteins were transferred to PVDF membrane and incubated with 5% of non-fat milk. The membranes were then incubated overnight at 4°C with primary antibody against human GGPPS1 (Proteintech, at a 1:200 dilution) or beta-actin (Boster, at a 1:1000 dilution). After being washed with PBST for 30 min, the membrane was probed with HRP-conjugated secondary antibody (Boster, at a 1:10000 dilution) for 1 h at room temperature. ECL detection reagent was used to demonstrate the results.

### Statistical analysis

Data were expressed as mean ± SD with the range given in parentheses, or categorized. Statistical comparisons were done using the Mann–Whitney U test, *t* test, ANOVA, or linear regression when data were normally distributed. Wilcoxon matched paired test was used to determine the significance of GGPPS1 expression among different kinds of liver tissues. χ2 test was performed to analyze the correlation between GGPPS1 expression and clinic pathological parameters. All statistical procedures were done using SAS (version 9.0; SAS, Inc.). Values of *p* < 0.05 were considered statistically significant.

## Results

### Characteristics of patients with HCC

In 70 patients (62 males and 8 females; median age, 54.09 years) who underwent curative resection (60 cases for regular hepatectomy and 10 for orthotopic liver transplantation), the average tumor size was 7.59 × 5.88 cm. Liver cirrhosis was detected in 42 patients, 9 patients had chronic hepatitis, and the remaining 19 patients without hepatitis or cirrhosis.

The etiologies of underlying liver diseases were hepatitis B in 42 patients, hepatitis C in 6 patients, and mixed viral infection in 3 patients. According to International Union Against Cancer recommendation, 15 patients were classified as stage I, 19 patients as stage II, 24 patients as stage III, and 12 patient as stage IV. Sixty-one patients were in child’s class A, 6 in class B and 3 in class C. The basic information of all the patients was attached in Additional file 1.

### Higher expression of GGPPS1 in tumor tissue comparing to adjacent nonmalignant tissues

**Figure 1:**
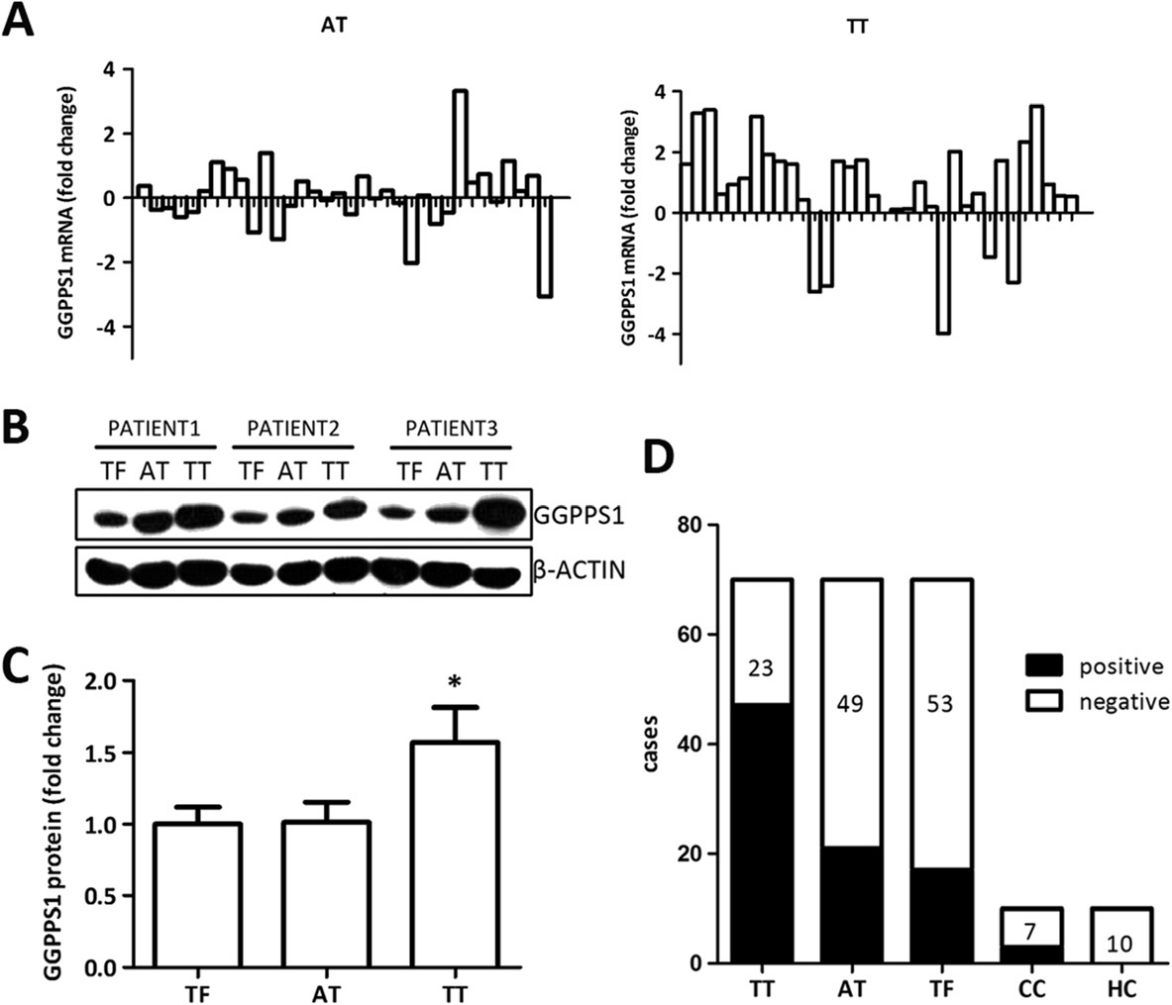
**Expression of GGPPS1 in liver tissues and tumor tissues of the patients with hepatocellular carcinoma. (A)** Relative expression level of GGPPS1 mRNA in 34 paired adjacent nonmalignant (AT) and tumor tissues (TT) comparing with tumor-free (TF) by qRT-PCR. Logarithm of fold change is shown. **(B)** Representative western blots analysis of GGPPS1 expression in 15 paired TF, AT and TT. **(C)** Semiquantitative western blots analysis of lysates of 15 paired tissue samples is shown. The mean protein expression level was significantly increased in TT compared with TF. **(D)** Immunohistochemical staining of anti-GGPPS1 antibody in paired AT and TT (n = 70), cirrhotic liver as cirrhosis control (CC, n = 10), and normal liver as healthy control (HC, n = 10) is shown.

To investigate the expression of GGPPS1 in HCC patients, Q-PCR was used to analyze the expression of GGPPS1 mRNA in 34 paired TT, AT and TF liver tissues. The results demonstrated that GGPPS1 mRNA was upregulated in 85.29% TT compared with TF and AT, and the mean relative level of GGPPS1 mRNA in TT and AT comparing to TF was 5.69 and 1.28 (Figure 1A). Consistently, western blots showed that protein level of GGPPS1 in TT was noticeably higher than that in TF and AT in 11 of the 15 patients (Figure 1B). However, there was no difference of GGPPS1 in mRNA and protein level between AT and TF (Figure 1C). To further confirm the results, immunohistochemistry was performed to assess the expression of GGPPS1. GGPPS1 was positively stained in 67.14% of TT, which was higher than that in AT (30%), TF (24.29%), CC (30%), and HC (zero) (Figure 1D, *p* < 0.001).

### GGPPS1 mainly distributes in the cytoplasm of liver tumor cells

**Figure 2:**
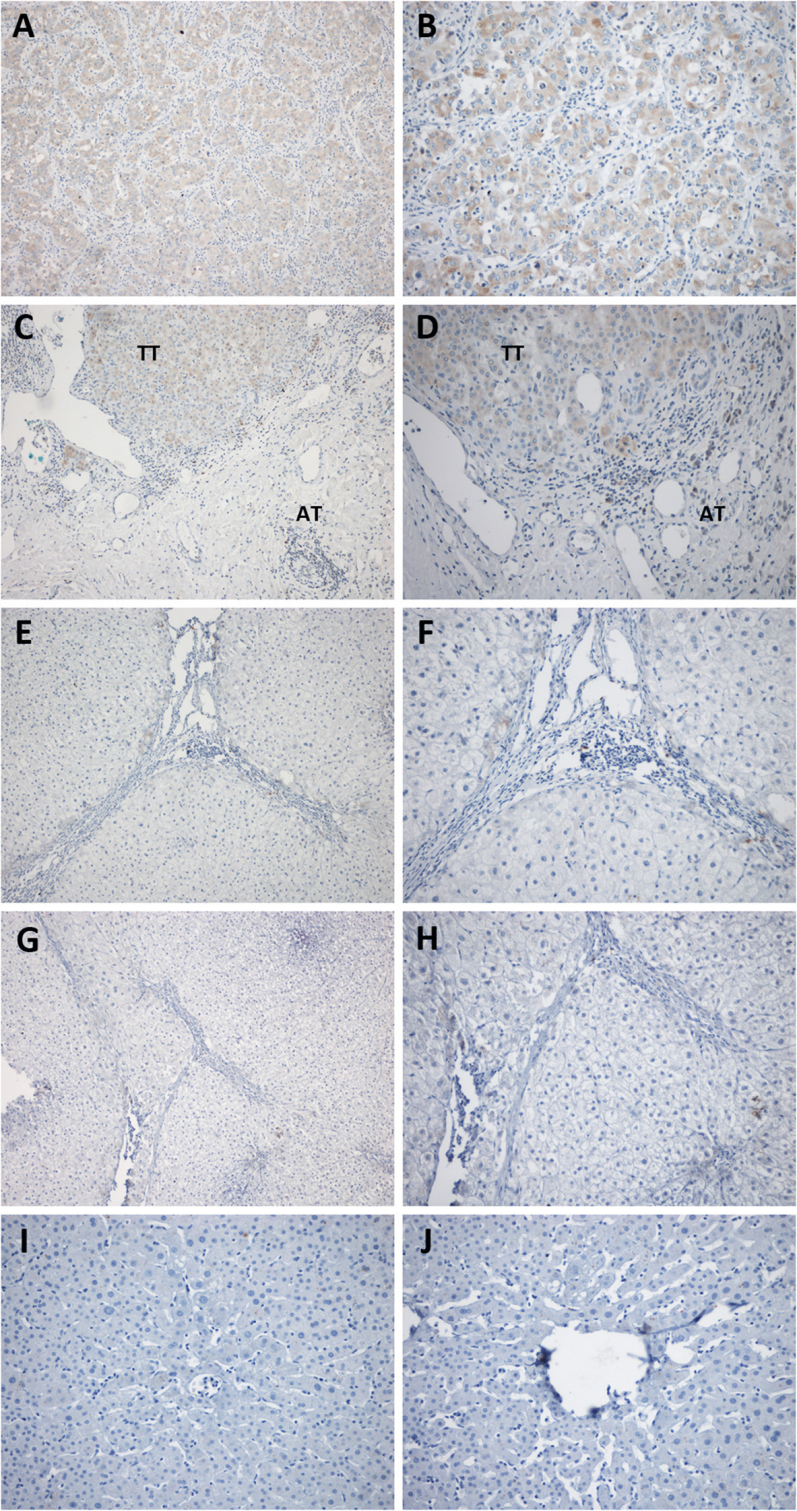
**Distribution of GGPPS1 antigen in liver tissues and tumor tissues of the patients with hepatocellular carcinoma.** The representative images are shown from tumor tissues (TT, **A** and **B**), adjacent non-tumor tissues (AT, **C** and **D**), tumor free tissues (TF, **E** and **F**), cirrhotic controls (CC, **G** and **H**), healthy controls (HC, **I** and **J**) tissues. Magnification: **A**, **C**, **E**, **G**, **I** 100×, **B**, **D**, **F**, **H**, and **J** 200 ×.

Our previous investigations reported that GGPPS1 was also found in airway epithelium, alveolar epithelial cells, and invasive inflammatory cells in cigarette smoke-related lungs [16]. The distribution and expression of GGPPS1 in paired TT, AT and TF (n = 70), cirrhotic liver as cirrhosis control (CC, n = 10), and normal liver as healthy control (HC, n = 10), was evaluated with immunohistochemistry. The positive staining of GGPPS1 mainly existed in cytoplasm of tumor cells (Figure 2A and B). Of note, GGPPS1 signals were less in the hepatocytes in AT and TF (Figure 2C and D, E and F). In cirrhotic liver or normal, GGPPS1 was negative or weakly positive (Figure 2G and H, I and J). Therefore, GGPPS1 mainly distributes in the cytoplasm of liver tumor cells.

### Expression of GGPPS1 in TT is associated with cirrhosis and GGPPS1 in AT

We checked the GGPPS1 distribution in TT and AT among the patients with or without cirrhosis or hepatitis. The proportion of GGPPS1-positive distribution in TT is higher in the patients with cirrhosis than in the patients without cirrhosis (*p* = 0.0484), while no statistic difference was found about GGPPS1 in TT between the patients with and without hepatitis (*p* = 0.4428) (Table 1). Positive correlation between the expression of GGPPS1 in TT and AT was also found. However, No statistic difference was found about GGPPS in AT between the patients with and without cirrhosis/hepatitis (*p* = 0.8314/0.1307).

**Table 1:** The correlation among cirrhosis, hepatitis and GGPPS1 antigen in tumor tissues and adjacent non-tumor tissues of the patients with HCC

**Variable**	**Category**	**GGPPST**		***p***	**GGPPSA**		***p***
		**Negative**	**Positive**		**Negative**	**Positive**	
**Cirrhosis**	**No**	13	15		20	8	
	**Yes**	10	32	0.0484	29	13	0.8314
**Hepatitis**	**No**	10	16		21	5	
	**Yes**	13	31	0.4428	28	16	0.1307
**GGPPSA**	**Negative**	17	32				
	**Positive**	6	15	<. 0001			

GGPPST represents GGPPS1 antigen in tumor tissues, and GGPPSA represents GGPPS1 antigen in adjacent non-tumor tissues.

### Expression of GGPPS1 is associated with biological character of HCC

To determine the clinical significance of GGPPS1 expression in HCC, the relationship between GGPPS1 expression and clinic pathological features was analyzed. Significant correlations were found between GGPPS1 expression and variables including TNM stage (*p* = 0.0059), T stage (*p* = 0.0041), vessel invaded (*p* = 0.0009), early recurrence (*p* = 0.0065), and recurrence (*p* = 0.0444) (Table 2). In a word, HCC patients with higher GGPPS1 expression had a higher tendency to be with advanced stage, vessel invaded, early recurrence, or recurrence. There were no statistical connections between GGPPS1 expression in TT and other clinic pathological parameters, such as age, gender, AFP, tumor differentiation, tumor necrosis, nodal number, CD34, p53, PCNA, VEGF, EGFR, or COX-2 (*p* > 0.05, Additional file 2). Moreover, there were no statistical correlations between GGPPS1 expression in AT and clinic pathological parameters, except gender (*p* > 0.05, Additional file 2).

**Table 2:** Clinic pathological factors and expression of GGPPS1 antigen in tumor tissues and adjacent non-tumor tissues of the patients with HCC

**Variables**	**Category**	**GGPPST**		***p***	**GGPPSA**		***p***
		**Negative**	**Positive**		**Negative**	**Positive**	
**AFP**	**<40**	12	23		23	12	
	**> = 40**	11	24	0.7991	26	9	0.4339
**Stage**	**1**	10	5		11	4	
	**2**	7	12		15	4	
	**3**	5	19		16	8	
	**4**	1	11	0.0059	7	5	0.6161
**Tumor**	**1**	10	5		11	4	
	**2**	7	11		13	5	
	**3**	5	19		17	7	
	**4**	1	12	0.0041	8	5	0.8951
**Vessel invaded**	**No**	18	17		27	8	
	**Yes**	5	30	0.0009	22	13	0.1922
**Early recurrence**	**No**	14	43		42	15	
	**Yes**	9	4	0.0065	7	6	0.1421
**Recurrence**	**No**	11	34		34	11	
	**Yes**	12	13	0.0444	15	10	0.1736

GGPPST represents GGPPS1 antigen in tumor tissues, and GGPPSA represents GGPPS1 antigen in adjacent non-tumor tissues.

## Discussion

Our study showed that GGPPS1 mRNA and protein expression levels were remarkably upregulated in HCC tissues, while it was much lower in paired adjacent nonmalignant and tumor free tissues and absent in healthy samples. Study of HCC epidemiology shows that more than 80% hepatitis B and C patients presenting with HCC are already cirrhotic [23]. In this study, though there was no statistic relationship between GGPPS1 and hepatitis, GGPPS1 was confirmed to be related with cirrhosis, pointing to the possibility that GGPPS1 might contribute to HCC through cirrhosis.

The characterization of genes that are differentially expressed between tumor and adjacent nonmalignant tissues is now considered meaningful in diagnosing the processes from normal to neoplastic. Immunohistochemistry results demonstrated that patients with higher GGPPS1 expression in TT had later pathology stage, which suggested that the upregulation of GGPPS1 could reflect lower differentiation and more aggressive clinical behavior in HCC. Therefore, GGPPS1 could act as a novel and useful biomarker reflecting incipient nodule and prevent deterioration from cirrhosis to HCC by surgical resection in early stage.

GGPPS is responsible for producing GGPP, and previous studies demonstrated that GGPPS1 was essential for prenylation modification of Ras [17]. Ras proteins are being studied intensively in many laboratories because Ras-Raf-MAPK cascades are key intracellular signaling pathways involved in the regulation of normal cell functions and also vital in pathological stress-exposure processes. Thus, any aberrant regulation of MAPK cascades may render cells insensitive to regulatory signals and contribute to tumor genesis [24]. In our study, both the expression of GGPPS1 mRNA and protein were dramatically upregulated in tumor tissues, and patients with higher expression of GGPPS1 showed advanced tumor stage and vessel invasion. We therefore hypothesized that aberrant prenylation of Ras could enhance liver carcinogenesis through overactivation of MAPK pathway. Meanwhile, in addition to Ras, another small G protein RhoA has also been reported to be involved in the progression and metastasis in HCC [25]. Clinic results strongly suggested that increased RhoA expression in HCC was correlated with vessel invasion and cell differentiation of HCC [26]. In our study, there was a sharp increase of GGPPS1 in patients with vessel invasion and later tumor stage, suggesting that upregulated GGPPS1 could elevate prenylated RhoA, further accelerating invasion and intrahepatic metastasis. Moreover, studies demonstrated that RhoA GTPase might regulate cell survival *via* Bcl2 family proteins in a p53-independent manner [27], indicating that higher expression of GGPPS1 could inhibit apoptosis and induce proliferation, therefore resulting in tumor genesis though a RhoA-mediated mechanism.

Recently, it has been demonstrated that LTsc1KO mice with hyperactive mTORC1 signaling display metabolic abnormalities and subsequently develop HCC [28,29]. Considering that activated Cdc42 and Rac1 potently stimulated S6K1 in vitro and in vivo [30], we hypothesize that aberrant protein prenylation caused by GGPPS1 may accelerate liver carcinogenesis by regulating Cdc42/Rac1 activation of mTORC1 and subsequent aberrant lipid homeostasis. However, our results showed no relation between GGPPS1 and CD34, p53, PCNA, VEGF, EGFR or COX-2, which were widely used in clinical pathology. The molecular basis for this observation remains to be elucidated.

## Conclusion

In summary, the present study revealed the role of GGPPS1 in HCC, further supporting the impact of prenylation during neoplastic development and progression. Notably, GGPPS1 showed significant correlation with pathology stage, cirrhosis, vessel invasion, and recurrence. This establishes feasibility of GGPPS1 in predicting biological character of HCC in patients with cirrhosis. Our results also indicated that disturbance of prenylation of Ras and Ras-related small GTP-binding proteins may serve as a therapeutic strategy. Once upstream and downstream signaling pathways are understood, these findings will be practical for prognosis and prevention of deterioration of incipient lesion.

## Competing interests

The authors declare that they have no competing interests.

## Authors’ contributions

DCY participated in its design, carried out western blot analysis and wrote the paper. JL collected all of specimens and clinical database, and carried out PCR and analyzed the results. JC carried out the immunoassays and analyzed the results, collected clinical database. JJS performed real-time PCR. CJL and HGX participated in its design. BX and YTD conceived of the study, participated in its design and give final approval of the version to be published. All authors read and approved the final manuscript.

## Pre-publication history

The pre-publication history for this paper can be accessed here:

http://www.biomedcentral.com/1471-2407/14/248/prepub

## Supplementary Material

Additional file 1: Table S1 Basic demographic and clinical characteristics of 70 HCC patients. Click here for file

Additional file 2: Table S2 Clinic pathological factors and expression of GGPPS1 antigen in tumor tissues and adjacent non-tumor tissues of the patients with HCC. Click here for file
