# Supplementary material for: GGPPS1 predicts the biological character of hepatocellular carcinoma in patients with cirrhosis
Source: BMC Cancer. 2014 Apr 9;14:248. doi: 10.1186/1471-2407-14-248 (PMC4028285; doi:10.1186/1471-2407-14-248)
Supplement: Additional file 2: Table S2 — Clinic pathological factors and expression of GGPPS1 antigen in tumor tissues and adjacent non-tumor tissues of the patients with HCC. [file 1471-2407-14-248-S2.docx]

Table S2. Clinic pathological factors and expression of GGPPS1 antigen in tumor tissues and adjacent non-tumor tissues of the patients with HCC

| Variables | Category | GGPPST | | *p* | GGPPSA | | *p* |
| --- | --- | --- | --- | --- | --- | --- | --- |
|  |  | **Negative** | **Positive** |  | **Negative** | **Positive** |  |
| Gender | **Female** | 3 | 5 |  | 8 | 0 |  |
|  | **Male** | 20 | 42 | 0.5257 | 41 | 21 | 0.0478 |
| Stage | **1** | 10 | 5 |  | 11 | 4 |  |
|  | **2** | 7 | 12 |  | 15 | 4 |  |
|  | **3** | 5 | 19 |  | 16 | 8 |  |
|  | **4** | 1 | 11 | 0.0059 | 7 | 5 | 0.6161 |
| T | **1** | 10 | 5 |  | 11 | 4 |  |
|  | **2** | 7 | 11 |  | 13 | 5 |  |
|  | **3** | 5 | 19 |  | 17 | 7 |  |
|  | **4** | 1 | 12 | 0.0041 | 8 | 5 | 0.8951 |
| Difference | **1** | 1 | 1 |  | 1 | 1 |  |
|  | **2** | 7 | 19 |  | 20 | 6 |  |
|  | **3** | 1 | 3 | 0.7772 | 4 | 0 | 0.3746 |
| Necrosis | **No** | 22 | 43 |  | 45 | 20 |  |
|  | **Yes** | 1 | 4 | 0.4657 | 4 | 1 | 0.8424 |
| vessel invaded | **No** | 18 | 17 |  | 27 | 8 |  |
|  | **Yes** | 5 | 30 | 0.0009 | 22 | 13 | 0.1922 |
| multiple   nodules | **No** | 9 | 15 |  | 17 | 7 |  |
|  | **Yes** | 14 | 32 | 0.5503 | 32 | 14 | 0.9125 |
| CD34 | **No** | 16 | 28 |  | 32 | 12 |  |
|  | **Yes** | 5 | 12 | 0.6084 | 10 | 7 | 0.2931 |
| COX2 | **Negative** | 4 | 13 |  | 14 | 3 |  |
|  | **Positive** | 19 | 32 | 0.3002 | 33 | 18 | 0.1726 |
| EGFR | **Negative** | 10 | 13 |  | 14 | 9 |  |
|  | **Positive** | 6 | 23 | 0.077 | 19 | 10 | 0.7296 |
| HBsAg | **Negative** | 2 | 8 |  | 8 | 2 |  |
|  | **Positive** | 11 | 18 | 0.9278 | 17 | 12 | 0.2049 |
| PCNA | **Negative** | 19 | 32 |  | 36 | 15 |  |
|  | **Positive** | 4 | 9 | 0.4633 | 9 | 4 | 0.5854 |
| P53 | **Negative** | 8 | 14 |  | 17 | 5 |  |
|  | **Positive** | 11 | 21 | 0.8805 | 18 | 14 | 0.1119 |
| VEGF | **Negative** | 3 | 6 |  | 6 | 4 |  |
|  | **Positive** | 15 | 19 | 0.0909 | 24 | 12 | 0.7806 |
| Early recurrence | **No** | 14 | 43 |  | 42 | 15 |  |
|  | **Yes** | 9 | 4 | 0.0065 | 7 | 6 | 0.1421 |
| Recurrence | **No** | 11 | 34 |  | 34 | 11 |  |
|  | **Yes** | 12 | 13 | 0.0444 | 15 | 10 | 0.1736 |
| Cirrhosis | **No** | 13 | 15 |  | 20 | 8 |  |
|  | **Yes** | 10 | 32 | 0.0484 | 29 | 13 | 0.8314 |
| Hepatitis | **No** | 10 | 16 |  | 21 | 5 |  |
|  | **Yes** | 13 | 31 | 0.4428 | 28 | 16 | 0.1307 |
| AFP | **<40** | 12 | 23 |  | 23 | 12 |  |
|  | **>=40** | 11 | 24 | 0.7991 | 26 | 9 | 0.4339 |
| GGPPSA | **Negative** | 17 | 32 |  |  |  |  |
|  | **Positive** | 6 | 15 | <.0001 |  |  |  |

*GGPPST represents GGPPS1 antigen in tumor tissues, and GGPPSA represents GGPPS1 antigen in adjacent non-tumor tissues.
